# Prophylactic action of ayahuasca in a non-human primate model of depressive-like behavior

**DOI:** 10.3389/fnbeh.2022.901425

**Published:** 2022-11-04

**Authors:** Maria Lara Porpino de Meiroz Grilo, Geovan Menezes de Sousa, Lilían Andrade Carlos de Mendonça, Bruno Lobão-Soares, Maria Bernardete Cordeiro de Sousa, Fernanda Palhano-Fontes, Draulio Barros de Araujo, Daniel Perkins, Jaime Eduardo Cecilio Hallak, Nicole Leite Galvão-Coelho

**Affiliations:** ^1^Laboratory of Hormone Measurement, Department of Physiology and Behavior, Federal University of Rio Grande do Norte (UFRN), Natal, RN, Brazil; ^2^Postgraduate Program in Psychobiology, Department of Physiology and Behavior, Federal University of Rio Grande do Norte (UFRN), Natal, RN, Brazil; ^3^Department of Biophysics and Pharmacology, UFRN, Natal, RN, Brazil; ^4^National Science and Technology Institute for Translational Medicine (INCT-TM), São Paulo, Brazil; ^5^Brain Institute, Federal University of Rio Grande do Norte (UFRN), Natal, RN, Brazil; ^6^School of Social and Political Science, University of Melbourne, Melbourne, VIC, Australia; ^7^Department of Neuroscience and Behavioral Sciences, University of São Paulo, São Paulo, Brazil; ^8^Department of Physiology and Behavior, UFRN, Natal, RN, Brazil; ^9^NICM Health Research Institute, Western Sydney University, Westmead, NSW, Australia

**Keywords:** ayahuasca, resilience, *Callithrix jacchus*, depression model, prophylaxis

## Abstract

Observational studies of long-term users of ayahuasca, an Amazonian psychedelic brew, suggest an increase in resilience *via* improvements in emotion and cognition. Ayahuasca has also demonstrated clinical antidepressant effects in human and animal studies; however, its potential prophylactic action in depression has not been previously studied. Therefore, this experimental study sought to evaluate the potential prophylactic effects of repeated and long-term ayahuasca use, *via* the modulation of resilience, in a non-human primate animal model, *Callithrix jacchus*, subjected to a protocol for induction of depressive-like behavior. For the formation of the study groups, some juvenile marmosets were kept in their family groups (GF = 7), while for the two experimental groups, the animals were removed from the family and kept socially isolated. Then, part of the isolated animals made up the group in which ayahuasca was administered (AG, *n* = 6), while for others, no intervention was made (IG, *n* = 5). AG animals took ayahuasca (1.67 mL/300g body weight) at weeks 4 (before isolation), 8, and 12 (during isolation) of the study. More adaptive stress response was observed for the AG when compared to the IG. The AG showed higher cortisol reactivity and fecal cortisol levels than IG, while both measures were similar to FG. Moreover, AG animals showed no signs of anhedonia and no increase in chronic stress-related behaviors, which were expressed by the IG. Thus, ayahuasca seems to promote the expression of resilient responses, indicating a prophylactic action, buffering the emergence of depressive-like behaviors and cortisol alterations associated with major depression. These results are encouraging for further research on the prophylactic use of psychedelics to prevent psychopathologies associated with chronic stress.

## Introduction

Major Depression Disorder (MDD) is strongly associated with chronic stress and a vulnerability phenotype (Ancelin et al., 2017; Teng et al., 2021). Vulnerability is the predisposition of neuropsychological networks to a dysfunctional state, leading to a non-adaptive stress response (Daskalakis et al., 2013; Borsboom, 2017). Resilience, on the other hand, is the ability to successfully adapt in face of adversity, which means returning to homeostatic conditions after stressful experiences. The processes that modulate resilience may be either innate or achieved during life (Russo et al., 2012; Borsboom, 2017; Cathomas et al., 2019). It is suggested that physical exercise (Silverman and Deuster, 2014), body-mind therapies (Galante et al., 2018; Nirwan et al., 2021), psychotherapies (Joyce et al., 2018), social support (Hunter et al., 2018), and some substances that act on stress response systems (Brachman et al., 2016) may act as resilience-promoting factors.

Other factors influencing the high prevalence of MDD include a partial response of some patients to antidepressant medications (Aherne et al., 2017; Hengartner and Plöder, 2018), delays in clinical improvement after commencing treatment, and adverse effects associated with drugs currently used, which may result in treatment drop-outs (Belzung et al., 2014). Therefore, great effort is being made to identify more effective treatments. In this context, studies of classic psychedelics with human and experimental models have received increased attention (Lysergic acid diethylamide, psilocybin, and ayahuasca: Galvão-Coelho et al., 2021; Lysergic acid diethylamide, dos Santos et al., 2018; Psilocybin: Goldberg et al., 2020; Ayahuasca: Palhano-Fontes et al., 2019).

To date, psychedelic studies for mental health conditions have employed these substances as treatments, not as prophylactic agents. However, based on the idea that resilience can be acquired (Joyce et al., 2018), protocols of prophylaxis for MDD using psychedelics should be further explored, since they may enable a reduction in the individual, social, and economic damage caused by this psychopathology (Price, 2016; Firth et al., 2019; Kočárová et al., 2021).

Some studies have tested the prophylactic effect of ketamine in reducing the incidence of psychopathologies during chronic stress situations. Ketamine is an anesthetic with low psychedelic effects, recently approved in some countries for treatment-resistant MDD (Hashimoto, 2019). The acute prophylactic use of ketamine has been shown to prevent the emergence of depressive and anxiety-like behaviors in rodents when exposed to psychosocial and inflammatory chronic stress protocols (Brachman et al., 2016; Mastrodonato et al., 2020). In addition, the acute prophylactic administration of ketamine also reduced the incidence of postpartum depression in women (Ma et al., 2019).

On the other hand, among the classic serotoninergic psychedelics (ayahuasca/*N, N*-dimethyltryptamine, psilocybin, lysergic acid diethylamide, and mescaline), no studies have reported on potential prophylactic effects (Krebs and Johansen, 2013; Johansen and Krebs, 2015; Bogenschutz and Ross, 2016). Ayahuasca is a psychedelic brew from the Amazon region, which is also used in some Brazilian syncretic religions. Long-term observational studies of religious users of ayahuasca have identified good mental health in the emotional and cognitive domains (Barbosa et al., 2009; Bouso et al., 2012). A recent large global online survey, with data from about 12,000 ayahuasca users, has confirmed these benefits in relation to affective symptoms and substance use (Perkins et al., 2021, 2022; Sarris et al., 2021). Moreover, some clinical trials and animal models studies have reported the antidepressant action of ayahuasca (de Osório et al., 2015; Sanches et al., 2016; Palhano-Fontes et al., 2019).

Ayahuasca can induce some mild acute side effects, such as increased blood pressure and heart rate, vomiting and nausea, as well as a brief increase in anxiety (Riba et al., 2001; dos Santos et al., 2012). However, the toxicity of ayahuasca is low (Bouso et al., 2011) and does not induce dose tolerance (Gable, 2007; Fábregas et al., 2010), making it an interesting substance to be tested in prophylactic protocols despite its acute side effects.

Ayahuasca is commonly made of two plants, *Banisteriopsis caapi*, containing reversible monoamine oxidase inhibitors, and *Psychotria viridis*, which contains *N, N*-dimethyltryptamine, the psychedelic substance that acts as 2A serotoninergic receptor agonist (Jacob and Presti, 2005; Cameron et al., 2019). These components of ayahuasca act on the main MDD neurotransmission systems. In addition, some studies have shown that ayahuasca acutely regulates hypothalamus–pituitary–adrenal (HPA) axis function, stimulates neuroplasticity processes, and reduces systemic inflammation, which is associated with an antidepressant action (de Menezes Galvão et al., 2018; da Silva et al., 2019; de Almeida et al., 2019; Galvão-Coelho et al., 2020).

The common marmoset, *Callithrix jacchus*, is an easy-to-handle small neotropical primate, which has a good adaptation to captivity and is currently listed in the category of Least Concern in the IUCN Red List of Threatened Species (Valença-Montenegro et al., 2021). Mainly because of their phylogenetic proximity with humans, common marmosets have been used in several studies of chronic stress, being a validated animal model of depression, both behaviorally and physiologically (Dettling et al., 2002; Pryce et al., 2002; Galväo-Coelho et al., 2008; Galvão-Coelho et al., 2017; da Silva et al., 2019; de Sousa et al., 2021). In addition, this primate has a well-defined ethogram (Stevenson and Poole's, 1976) and non-invasive techniques for measuring steroid hormones in feces (de Sousa and Ziegler, 1998), which makes it an efficient animal model in the study of psychopathologies associated with chronic stress (de Sousa et al., 2021).

Previous studies showed that chronic social isolation (between 8 and 13 weeks) has been validated as a protocol inducing depression and hypocortisolemia in juvenile marmosets (Galvão-Coelho et al., 2017; da Silva et al., 2019). However, it is important to note that the physiological and behavioral response to social isolation depends on the age and sex of the animal, as well as the duration of the stressor. Shorter periods of social isolation, that is, up to 4 weeks, generally induce an increase in cortisol levels (de Sousa et al., 2015) and are used as a protocol to induce a state of anxiety (Barros and Tomaz, 2002).

Given the advantage of validating protocols that prevent the incidence of MDD, especially in chronic stress, and the potential therapeutic and protective effects of ayahuasca, the potential prophylactic effects of ayahuasca in the emergence of a typical depressive state in marmosets were evaluated during the use of a protocol for induction of depressive-like behavior. It was hypothesized that the prophylactic use of ayahuasca will increase resilience in juvenile marmosets and prevent the onset of fecal cortisol changes and the emergence of depressive-like behaviors.

## Methods

### Animals and maintenance conditions

This study used 18 juvenile *Callithrix jacchus* males, aged between 7 and 9 months (within the juvenile II stage of development, according to de Castro Leão et al., 2009, who used an algorithm based on body weight for ontogenetic classification). A table with the ages of all animals is provided in Supplementary Table S1, and the median ages and body weights of each group at the beginning of the study are provided in Supplementary Table S2.

The animals were housed at the Primatology Center of the Universidade Federal do Rio Grande do Norte, which has followed the standards of the National Council for the Control of Animal Experimentation (law No. 11,749 of October 08, 2008, entitled “Lei Arouca”) and international standards of the Animal Behavior Society and International Primatological Society. The experimental protocols of this study were approved by the Ethics Committee on Animal Use according to protocol 032/2014 and addendum No. 5, 6, and 7.

The animals were fed twice a day, in the early morning and late afternoon, and the diet was based on a specific small animal feed, including some tropical fruits, eggs, sweet potato, banana, and cereals, supplemented with a multivitamin complex and glycogen. The animals were taken care of by a veterinarian. The breeding house kept the animals in natural conditions of light, temperature, and humidity. The animals in family groups were housed in two cages measuring approximately 3.0 × 2.0 × 1.0 m each, connected by a door that allowed them to pass through, while the socially isolated animals were kept in single cages with dimensions of 3.0 × 2.0 × 1.0 m. All cages are made of masonry, with a one-way glass window on one wall, where behavioral observations were made, and on the opposite wall, there was a barred door where water (*ad libitum*) and food were served. The cages had a grid roof that allowed animals to be exposed to the outside environment receiving natural light, humidity, and temperature of a tropical climate. The cages were enriched with branches of natural trees, wooden swings, and a nest box for the rest and comfort of the animal.

### Study design

**Figure 1:**
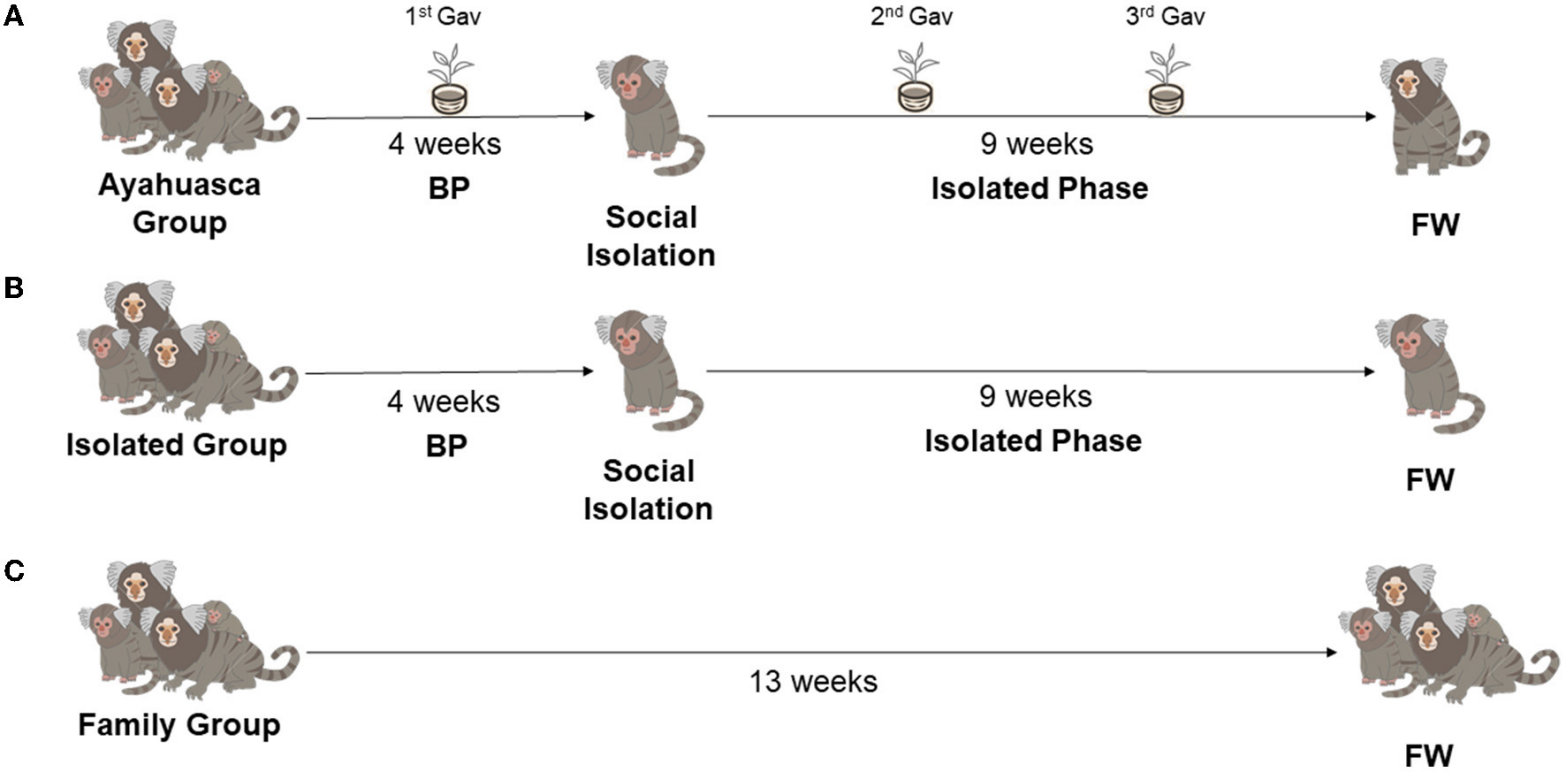
Study groups and phases. **(A)** Ayahuasca Group (AG Group; *n* = 6), the baseline phase (BP) is 4 weeks of every other day of data collection, which was followed by social isolation stress for depression induction (isolated phase- 9 weeks). This group had three ayahuasca gavages: 1st Gav (27th day of BP); 2nd and 3rd Gav (along isolated phase, being 25 days after the previous administration). **(B)** Isolated Group (IG; *n* = 5), animals that were submitted to these same study phases, however, did not receive the ayahuasca prophylactic intervention. **(C)** Family Group (FG; *n* = 7), the animals remained in their family group for 13 weeks without any intervention. FW is the final week of the study with daily data collection.

The design was based on previous studies that have validated and tested the induction of depressive-like behavior in juvenile marmosets through chronic social isolation (Galvão-Coelho et al., 2017; da Silva et al., 2019) (Figure 1).

Eighteen animals were randomly divided into three groups: In the first, the Ayahuasca Group (AG, *n* = 6), the animals were in their family groups for 4 weeks, a Baseline Phase (BP), when behavioral and endocrine (fecal cortisol) profiles were recorded. After that, the animals were socially isolated for 9 weeks (Isolated Phase), a protocol for inducing a depression-like behavior state. During the isolation phase, the animals did not have visual contact with conspecifics, but auditory and olfactory contact was possible. Moreover, there was no spatial constraint during the isolated phase. On the last baseline week, 3 days before isolation, these animals received the first prophylactic administration of ayahuasca *via gavage* (1st gavage), then two more gavages were made 25 and 50 days after the 1st gavage (totaling three gavages throughout the study). Gavage is a common veterinary technique used in marmosets and other animals, where the animal is immobilized by the veterinarian and a liquid is introduced into the stomach through a flexible tube placed in the mouth by a second person.

During the final week (FW) of the isolation phase (9th week), feces and behavior were recorded daily. In the second group, the Isolated Group (IG, *n* = 5), the animals were submitted to the same study phases of AG, however, did not receive the ayahuasca prophylactic intervention. In the third group, Family Group (FG *n* = 7), the animals remained in their family groups throughout the study period (13 weeks) without any intervention, i.e., without a depression protocol or ayahuasca administration. During the baseline phase and in the FW, the behavioral and endocrine (fecal cortisol) profiles were recorded. For all groups (AG, IG, and FG), behavioral data were recorded on alternate days in BP and daily in FW (Figure 1). Fecal sampling for cortisol measurement was performed on the same days of behavior observation. The behavioral and fecal collections were always done around 7:00 am, to avoid circadian variations (Ferreira Raminelli et al., 2001).

### Ayahuasca brew

The ayahuasca brew was donated for this study free of charge by Barquinha Temple, an ayahuasca religious institution located in Ji-Paraná/Roraima Brazil. The same batch was used throughout the study and for all animals. The brew is composed of 50% *B. caapi* bark and 50% *P. viridis* leaves, both boiled in water (100°C) for 60 h. The final solution was stored in glass bottles in the refrigerator. The main compounds in ayahuasca were measured using mass spectrometry resulting in the following values: 0.36 ± 0.01 mg/mL DMT, 1.86 ± 0.11 mg/mL harmine, 0.24 ± 0.03 mg/mL harmaline and 0.20 ± 0.05 mg/mL tetrahydroharmine. The ayahuasca solution was stored in the refrigerator at a temperature near 4°C.

The administered dose was 1.67 mL/300 g of marmoset body weight. The marmoset dose was initially based on the dose used in Brazilian religious ceremonies; there is around 1 mL of ayahuasca/kg. However, we adapted the human dose to the physiological characteristics of the marmoset using an allometric scale that considers metabolic differences between species and was described by Pachaly (2006): (1) basal metabolic indices of humans of a common 70 kg individual, 70 × 70^0.75^ = 1.694 kCal; (2) basal metabolic indices of the common marmoset (300 g), 70 × 0.3^0.75^ = 28.38 kCal; (3) dose of ayahuasca in mL per kCal, 100 mL/1.694 kCal = 0.06 mL/kCal; and (4) equivalent dose for a common marmoset, 0.06 mL/kCal × 28.35 kCal = 1.67 mL.

The frequency of ayahuasca administration used in this study was based on an average frequency of ayahuasca consumption in Brazilian religious rituals and psychedelic-based psychotherapy models (Carhart-Harris et al., 2021). The administration of ayahuasca was performed by veterinarians and researchers *via* gavage (da Silva et al., 2019).

### Behaviors

The behaviors of each animal were recorded by the continuous focal method, in which the observer records all the animal's behaviors simultaneously and uninterruptedly during a defined time window. Therefore, in contexts where the animal exhibits two or more behaviors at the same time, both are recorded. It should be noticed that when animals were with their family, the access to and time spent at the spout bottle used to evaluate sucrose intake (see below) could be limited due to the presence of the other animals. Observation sessions lasted 30 min when the frequency and duration of behaviors were recorded based on Stevenson and Poole's (1976) ethogram. The behaviors had their meaning adapted to the stress context (Galvão-Coelho et al., 2017) (Table 1). The anhedonia, i.e., the inability to feel pleasure, was assessed by the time (in seconds) spent ingesting a 4.16% aqueous sucrose solution (da Silva et al., 2019), provided in a spout bottle, only on test day and during the behavioral observation (30 minutes), when the animal had the choice whether it wanted to drink the water that was in the cage, and it was given *ad libitum* or the inserted bottle of sucrose. The reduction of ingestion over time, along the weeks of study, to lower levels than FG, was considered anhedonia (Galvão-Coelho et al., 2017) (Table 1).

**Table 1:** Behaviors based on Stevenson and Poole's (1976) ethogram adapted to the stress context (Galvão-Coelho et al., 2017).

**Behavior**	**Description**	**Stress context**
Scratching	The act of using the hands to rub some region of the body.	It is considered to be stereotyped behavior and an expression of anxiety.
Scent marking	The act of rubbing the anogenital or suprapubic region on a substrate.	Expression of anxiety
Autogrooming	The act of self-grooming	Acts like a stress/tension reducer
Food ingestion	The act of taking the piece of food to the mouth and ingesting it.	-
Locomotion	The act of moving between the different quadrants marked off in a cage.	Anxiety expression
Individual piloerection	The act of raising the hair and standing with the back arched.	Expression of sympathetic nervous system activation; acute stress response
Ingestion of the aqueous sucrose solution	The act of ingesting a palatable substance of water and sucrose in a spout bottle.	Measurement of the ability to feel pleasure; reduction is indicative of anhedonia

### Fecal cortisol

In a previous study from our laboratory, it was shown that male marmosets defecate more frequently in the first two 2-h intervals in the morning and the frequencies observed during these intervals (5:00–7:00 and 7:00–9:00 h) were significantly different from all remaining intervals (Castro and Sousa, 2005). Therefore, for all animals, in groups or isolated, the researcher observed the animals within this interval (between 07:00 and 08:00 h) through a unidirectional mirror until defecation occurred and then the collection of feces was performed using a disposable wooden spatula. Each sample was stored in 5 mL plastic tubes (Eppendorf), with the identification of the animal and the date. The tubes were then frozen and stored at around −10°C until cortisol assays. Fecal cortisol was measured by the competitive ELISA technique according to Munro and Stabenfeldt (1984), and adapted by de Sousa and Ziegler (1998). It consists of two steps: steroid extraction and cortisol dosage. The steroid extraction is made by hydrolysis and then methanol-ethanol solvolysis. The ELISA dosage step starts with the 96-well plate sensibilization with an anti-cortisol antibody (donation of Primate Center's Assay Services Laboratory, University of Wisconsin, USA), followed by a sandwich ELISA using a secondary anti-cortisol antibody (donation of Primate Center's Assay Services Laboratory, University of Wisconsin, USA) conjugated to a horseradish peroxidase enzyme, and the substrate for the color reaction was 2,2'-azino-bis[3-ethylbenzothiazoline-6-sulphonic acid], of which oxidation yields a green product—the readout of the spectrophotometer. The intra- and inter-assay coefficients of variation were 1.69 and 9.96%, respectively. Both are within the acceptable range, which means that the measures are reliable (Thomsson et al., 2014; Andreasson et al., 2015; Marcelletti et al., 2015).

### Statistical analysis

The quantitative dependent variables in this study are behavior and fecal cortisol data. Since there is large variability across individuals in behavioral and cortisol responses to social isolation (Galväo-Coelho et al., 2008; Galvão-Coelho et al., 2012; de Sousa et al., 2015), we have used in statistical analysis the percentage of variation of cortisol and behaviors between baseline and the last week of study (week 9), named reactivity. The categorical independent variables are the groups (FG, IG, and AG).

Reactivity for each subject was calculated as follows: ([∑ *FW* − ∑ *BP*]^*^100) / ∑ *BP*, and cortisol by $\left({\left[M_{d}\left(FW\right)-M_{d}\left(BP\right)\right]}^{*}100\right)/M_{d}\left(BP\right)$, where *M*_*d*_ is the median.

We have used, in the descriptive statistics, the median as a measure of central tendency and interquartile range, which shows the spread of the middle half of the distribution to the second or third quartiles. There is supplementary material (Supplementary Table S2) containing the values of central tendency and variability for all behaviors and cortisol by study phase.

The Boruta algorithm was used to select which behaviors have shown the greatest strength in distinguishing between the three groups (AG, FG, and IG). The Boruta test is based on the random-forest classifier. This algorithm works by creating noise “variables” by shuffling the original data into three levels—high, medium, and low (Maximal, Mean, and Minimal, respectively), to be used as a parameter for evaluating the importance of the dependent variables in group discrimination. Variables are considered important when their score (I) is greater than Shadow Max and are in an area of indecision when the score is very close to Shadow Max (Kursa and Rudnicki, 2010). For the behaviors classified by Boruta as important or in indecision shadow, and cortisol data, the nonparametric multivariate Kruskal-Wallis test, and the Mann-Whitney as a *posthoc* test, were applied to compare the reactivity between the groups.

Moreover, the effect size is reported as η^2^ for Kruskal-Wallis and *r* for the Mann-Whitney test. The η^2^ effect size was calculated as $\eta^{2}\;=\;\frac{H-k+1}{n-k}$, where *H, k*, and *n* stand for H-statistics, the number of independent variables, and the number of observations, respectively. The *r* effect size was calculated as $r\;=\;\frac{Z}{\sqrt{n}}$, where *Z* is the *Z*-statistic from the *posthoc* test and *n* is the number of observations.

For all tests, the significance level considered was *p* ≤ 0.05 (two-tailed).

## Results

### Behaviors

**Figure 2:**
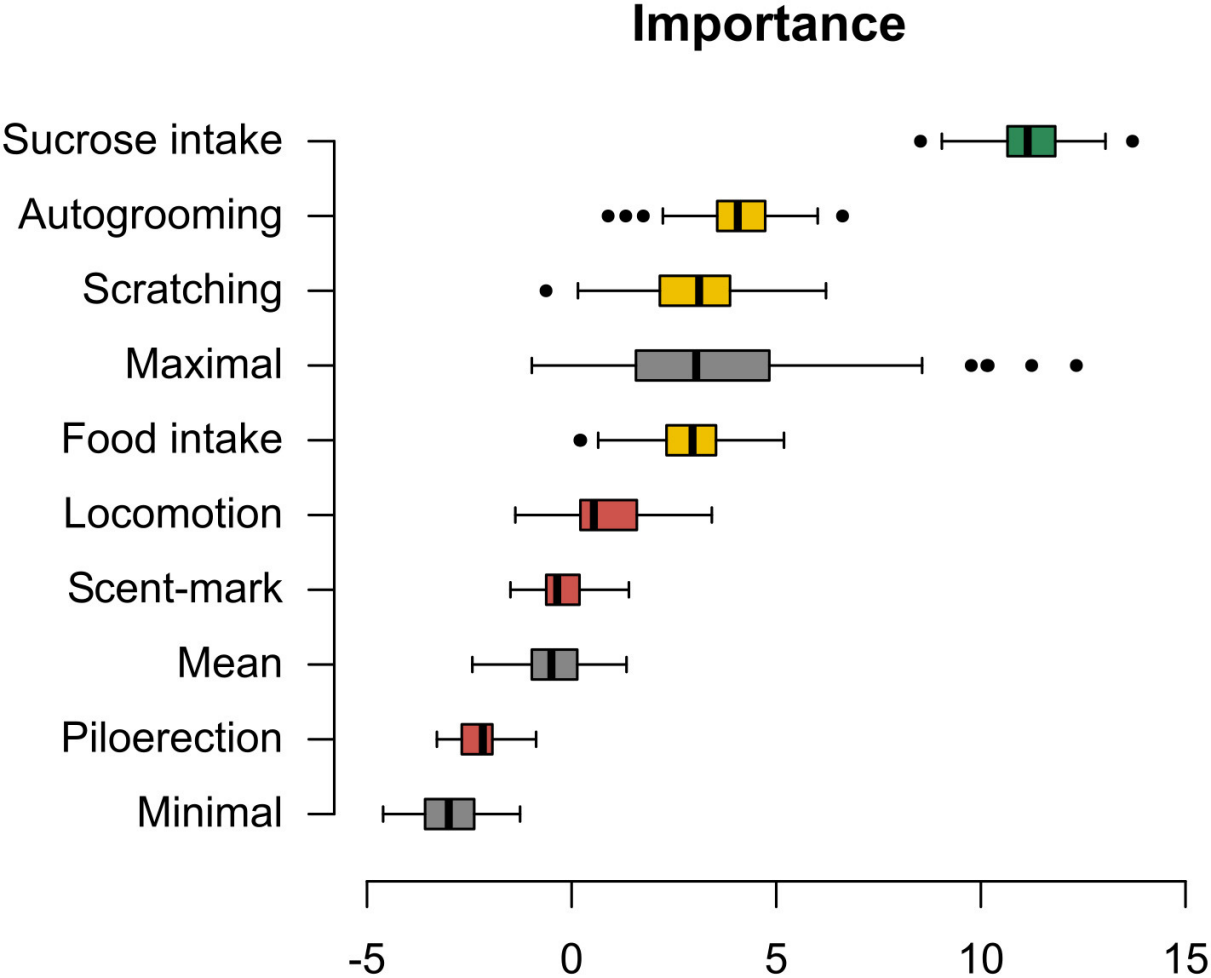
Boruta algorithm. Importance of behaviors for group discrimination as assessed by the Boruta algorithm. Green boxplots: important variables; yellow boxplots: undecided relevance; red boxplots: not important variables; gray boxplots: shuffled data at three levels of importance—high importance (low shuffle), mean importance (moderate shuffle), and low importance (high shuffle); black dots: outlier. The algorithm uses such shuffled data to decide on the importance classification. The score of importance is given on the *x*-axis.

The time spent ingesting the sucrose solution was the most relevant behavior for group discrimination (between the three groups: AG, FG, and IG), while autogrooming, scratching, and food ingestion frequencies were in the indecision shadow. The other behaviors were classified as noise, and therefore they were excluded from subsequent analyses (Figure 2; Supplementary Table S3). Baseline comparisons between groups for the selected variables are depicted in Supplementary Table S4.

**Figure 3:**
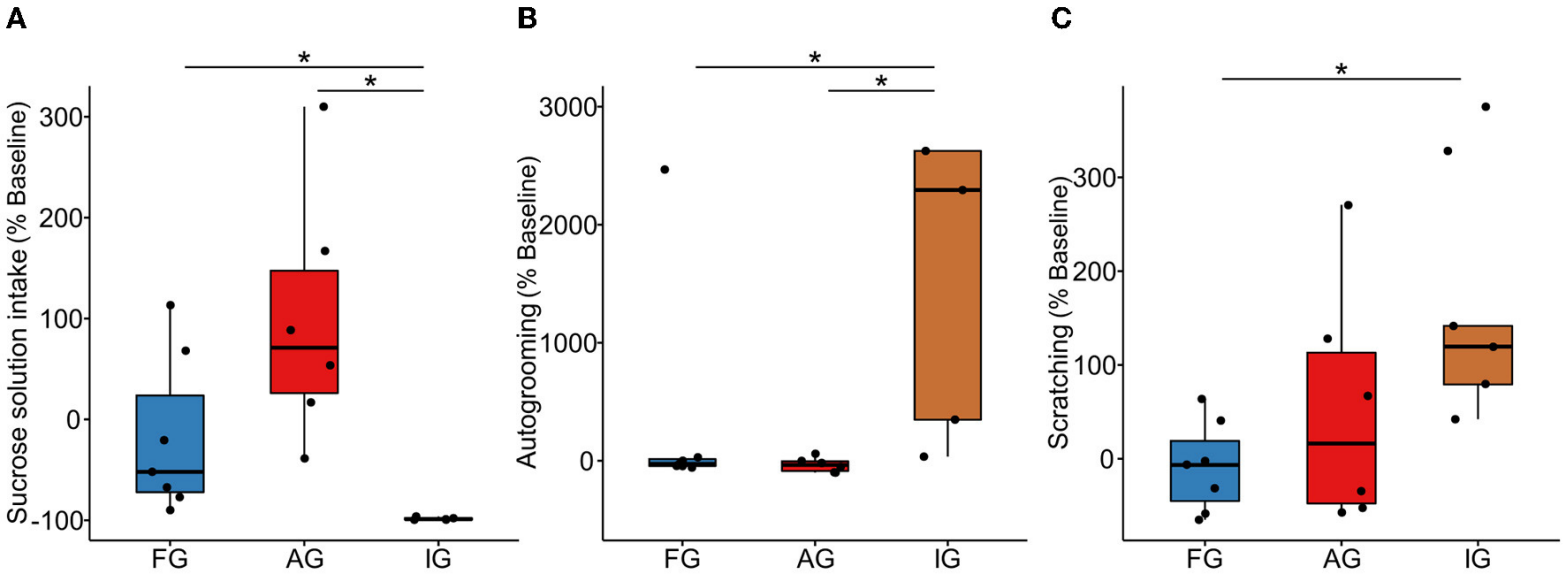
Box plot of behavioral reactivity. **(A)** Sucrose solution ingestion, **(B)** Autogrooming, and **(C)** Scratching. Ayahuasca group (AG, *n* = 6), isolated group (IG, *n* = 5), and family group (FG, *n* = 7); each black dot represents an individual. *Kruskal Wallis non-parametric multivariate test, and Mann-Whitney *posthoc* test, *p* ≤ 0,05. %: indicates the reactivity, showing the difference in the individual behavior of the final week compared to the baseline phase ([∑ *FW* − ∑ *BP*]*100) / ∑ *BP*.

The ingestion of sucrose solution (*H*_(2,17)_ = 12.09, *p* = 0.002, η^2^ = 0.673), autogrooming (*H*_(2,17)_ = 8.33, *p* = 0.015, η^2^ = 0.423), and scratching (*H*_(2,17)_ = 6.41; p = 0.040, η^2^ = 0.295) were different between the three groups, but not the food ingestion (Feeding: *H*_(2,17)_ = 3.87, *p* = 0.144, η^2^ = 0.125). The AG (median [interquartile range] = 71.08 [199.78]) showed a higher sucrose solution intake compared with IG (−98.99 [2.46]; *U* = 0.000, *p* = 0.006, *r* = 0.826), while AG and FG presented similar levels of intake (*U* = 8.00, *p* = 0.063, *r* = 0.515). The sucrose intake was significantly lower for IG when compared to FG (−52.00 [145.14]; *U* = 0.00, *p* = 0.004, *r* = 0.820) (Figure 3A). The AG (−34.73 [113.26]) presented lower autogrooming reactivity than IG (2,294.29 [38,680.72]; *U* = 1.00, *p* = 0.011, *r* = 0.771) and similar to FG (−26.07 [74.89]; *U* = 15.50, *p* = 0.431, *r* = 0.218). IG also had higher reactivity than FG (*U* = 3.00, *p* = 0.019, *r* = 0.680) (Figure 3B). Moreover, AG (16.23 [217.29]) showed similar scratching reactivity to the IG (119.58 [197.80]; *U* = 8.00, *p* = 0.201, *r* = 0.385) and FG (−6.57 [99.78]; *U* = 15.00, *p* = 0.391, *r* = 0.238). On the other hand, the IG showed higher scratching reactivity than FG (*U* = 1.00, *p* = 0.007, *r* = 0.774) (Figure 3C).

### Fecal cortisol

**Figure 4:**
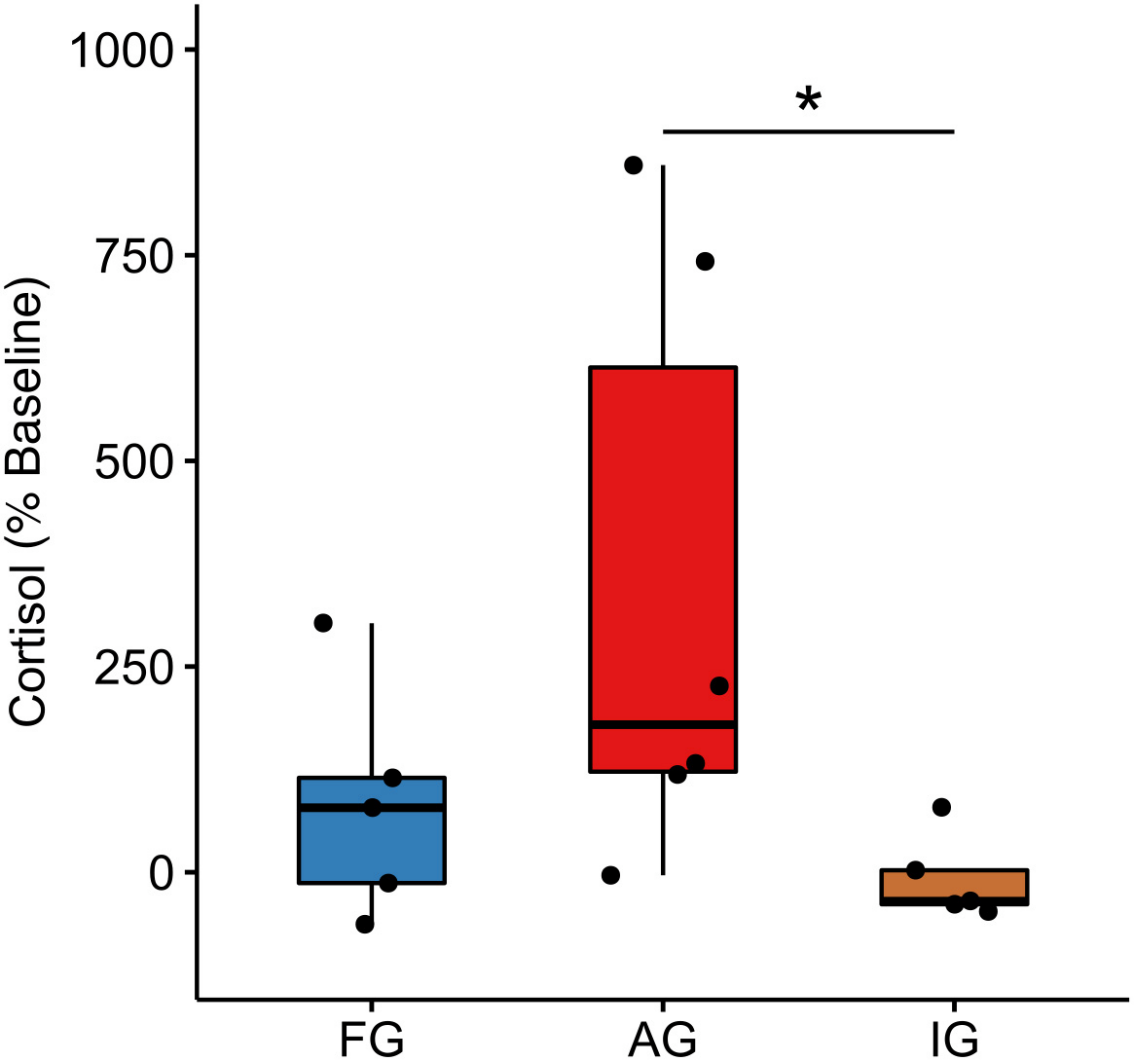
Fecal cortisol reactivity. Ayahuasca group (AG, *n* = 6), isolated group (IG, *n* = 5), and family group (FG *n* = 7); each black dot represents an individual. *Kruskal Wallis non-parametric multivariate test *p* ≤ 0,05. %: Indicates the reactivity, showing the difference in the individual cortisol levels of the final week compared to the baseline phase. ([*M*_*d*_(*FW*) − *M*_*d*_(*BP*)]*100) / *M*_*d*_(*BP*).

Regarding fecal cortisol (${\text{H}}_{\left(2,15\right)}=6.44,\text{p}=0.04,\eta^{2}=0.342$), the AG (179.00 [491.00]) showed a higher cortisol reactivity than IG (−35.10[41.30], *U*(10) = 2.00;*p* = 0.018, *r* = 0.716) and no difference with respect to FG (78.40[128.00];*U*(10) = 6.00, *p* = 0.100, *r* = 0.495). FG reactivity did not differ from IG (*U*(9) = 8.00, *p* = 0.347, *r* = 0.297) (Figure 4). Despite the lack of significance in cortisol reactivity between FG and IG, the effect size of the cortisol levels at FW between these groups is r = 0.628 (FG: 4.55 [1.64]; IG: 2.06 [1.00]), which falls within the large effect size window found in a previous study (Galvão-Coelho et al., 2017: *d* = 1.252 ~ *r* = 0.531).

## Discussion

We observed an apparent prophylactic action associated with ayahuasca administration *via* increased resilience, avoiding depressive-like behaviors, and stimulating cortisol reactivity during chronic social isolation. Juvenile males of *C. jacchus* administered with ayahuasca demonstrated a more adaptive stress response than isolated animals without this intervention, as well as behavioral reactivity and fecal cortisol levels similar to those of animals living in their families.

In animal models, anhedonia has been evaluated through the test of sucrose solution intake. Decreased intake of this solution (to levels below usual food adaption reductions) is characteristic of anhedonia (Der-Avakian and Markou, 2012; Willard and Shively, 2012; Galvão-Coelho et al., 2017). In the present study, this parameter most strongly differentiated the groups, while food ingestion, piloerection, scent marking, and locomotion behaviors did not significantly discriminate between groups (Pic-Taylor et al., 2015). The IG animals showed a stronger reduction in sucrose ingestion than FG, thus characterizing anhedonia, while AG had similar reactivity to FG marmosets, suggesting a prophylactic action of ayahuasca. A study with the same model and protocol observed that an acute dose of ayahuasca was not sufficient to reverse anhedonia (da Silva et al., 2019). Investigations into ketamine's use as a prophylactic, using mice, had limitations because they did not analyze anhedonia, which is a key feature in translational models of depression (Brachman et al., 2016; Mastrodonato et al., 2020). Although it is important to highlight that ketamine has demonstrated positive effects in reducing anhedonia in patients and some animal models of depression when this symptom is already present (Nogo et al., 2022).

In marmosets, autogrooming is a stress-reduction behavior (Neumann et al., 2001; Burkett et al., 2016). In this study, the AG showed lower reactivity to autogrooming when compared to the IG, and similar to FG. Thus, ayahuasca might be mediating resilience growth through a complementary mechanism to autogrooming. A previous study with marmosets and ayahuasca did not observe modulation of autogrooming (da Silva et al., 2019).

Scratching is indicative of anxiety (di Sorrentino et al., 2012). Ayahuasca-treated animals showed a similar scratching reactivity to isolated and family-living animals. FG had lower reactivity than isolated control groups, which probably resulted from the social interaction that buffers the stress response and has a protective effect against anxious signs (da Silva et al., 2019). Despite this result indicating slight protection of ayahuasca in favor of scratching prevention, the similarity between AG and IG requires further investigations, since an antidepressant effect of an acute dose of ayahuasca on reducing scratching in marmosets was previously observed (da Silva et al., 2019). Studies of a prophylactic ketamine regimen also observed a protective effect against anxious behaviors in rodents (Brachman et al., 2016; Mastrodonato et al., 2020).

Cortisol is one of the main molecular biomarkers of major depression, and changes in hypo and hypercortisolemia are both observed in patients (de Menezes Galvão et al., 2018, 2021; Varela et al., 2021). In this study, marmosets under ayahuasca showed a greater cortisol reactivity compared to animals in the isolated control group, thus avoiding HPA axis blunting and hypocortisolemia, observed in this last group and is usually seen as a result of this protocol (Galvão-Coelho et al., 2017; da Silva et al., 2019). In the final week, AG animals had similar cortisol levels to the family control group, which is important because it suggests that ayahuasca-induced cortisol reactivity does not lead to a maintained hypercortisolemia. Another factor that must be considered is the great variability of cortisol reactivity in the GA group, which may be a result of individual and environmental factors that modulate the stress response to isolation, such as basal cortisol levels and coping style (Galväo-Coelho et al., 2008; Galvão-Coelho et al., 2012; Quah et al., 2020) and the sex and age of the animals (de Sousa et al., 2015). Moreover, it also results from variability in the way cortisol responds to ayahuasca. Studies with marmosets and humans have pointed to cortisol rises in response to an acute dose of ayahuasca (de Menezes Galvão et al., 2018; da Silva et al., 2019). However, to date, this is the first study to investigate the long-term action of ayahuasca on cortisol.

It is important to emphasize the relevance of using juvenile animals in this study. Prophylactic therapies, such as the one tested here, would be of great benefit to adolescents during challenging life events; first, because adolescence is considered a critical window for social and neural plasticity (Larsen and Luna, 2018), and the incidence and recurrence rates of MD has been increasing at this age (Aalto-Setälä et al., 2002; Young, 2012; Breslau et al., 2017). An observational study comparing adolescent ayahuasca users and non-users found fewer psychiatric symptoms among users, suggesting a protective effect of ayahuasca (Da Silveira et al., 2005).

Despite our encouraging results, some limitations should be considered. The absence of marmoset females is an important limitation, mainly due to the higher incidence of depression in women (McHenry et al., 2014; Eid et al., 2019). The lack of sham gavages (i.e., with saline or water as a vehicle) in the stressed control group, happened mainly due to the restriction of confinement during the SARS-COVID-2 pandemic and led to some changes in the initial study methodology. Furthermore, although studies using non-human primates as animal models generally have a limited sample size (Hamadjida et al., 2018; Teng et al., 2021), the low number of animals may interfere with the interpretation of statistical results and make them not fully generalizable. Other factors can be considered, such as preferential access and competition for preferred foods in sucrose intake in the non-isolated control group, individual differences in innate coping strategies, and natural changes due to shifts in developmental stages. Finally, the application of a single intervention schedule (dose and frequency) is also a limitation. Thus, for future studies, a higher sample size with the inclusion of females and different age groups, as well as a protocol with sham gavages in the stressed group and different doses and frequencies of treatment with ayahuasca are recommended. Furthermore, despite the stability of the juvenile II stage, investigations considering a month-to-month age could make the data more accurate. This is particularly important because the juvenile stage is a phase of transition and transformation, although such changes are characteristic of adolescent animal models, and are also present when studying mental disorders in the human species.

To our knowledge this is the first experimental study that has tested a classic psychedelic in a prophylactic regimen, that is, an intervention applied before the onset of some symptoms and attenuating their onset in situations of chronic stress. The evidence presented could stimulate a promising new area of human research with classic psychedelics.

## Data availability statement

The raw data supporting the conclusions of this article will be made available by the authors, without undue reservation.

## Ethics statement

The animal study was reviewed and approved by Ethics Committee on Animal Use of Federal University of Rio Grande do Norte (protocol 032/2014 and addendum No. 5, 6, and 7).

## Author contributions

NG-C and MMG designed the experiment and wrote the first version of the manuscript. MMG collected the data. MMG and GS performed data analysis. All authors have reviewed, edited, and approved the manuscript.

## Funding

This study was financed in part by the Coordenação de Aperfeiçoamento de Pessoal de Nível Superior – Brasil (CAPES) – Finance Code 001. MMG was supported by CAPES (Higher Education Improvement Coordination, Proc. 88887.357778/2019-00). The National Science and Technology Institute for Translational Medicine (INCT-TM Fapesp 2014/50891-1; CNPq 465458/2014-9).

## Conflict of interest

The authors declare that the research was conducted in the absence of any commercial or financial relationships that could be construed as a potential conflict of interest.

## Publisher's note

All claims expressed in this article are solely those of the authors and do not necessarily represent those of their affiliated organizations, or those of the publisher, the editors and the reviewers. Any product that may be evaluated in this article, or claim that may be made by its manufacturer, is not guaranteed or endorsed by the publisher.
