# Supplementary material for: Prophylactic action of ayahuasca in a non-human primate model of depressive-like behavior
Source: Front Behav Neurosci. 2022 Nov 4;16:901425. doi: 10.3389/fnbeh.2022.901425 (PMC9672345; doi:10.3389/fnbeh.2022.901425)
Supplement: Supplementary file 1 [file Table_1.pdf]

Supplementary material

Table S1. Ages and body weight by group at beginning of study.

Group	Animal	Age (months)	Weight (g)
Familiar	1255	8	225.5
	1269	7	273
	1271	7	193
	1279	7	228
	1287	7	289.5
	2808	7	276
	2820	7	268
Isolated	1261	7	241.5
	1259	7	188.5
	1263	7	257
	1273	7	237
	1275	7	264
Ayahuasca	1407	9	228
	1409	9	240
	1419	8	236
	1417	7	250
	Ex05	8	254
	Ex07	8	238

All animals were within the juvenile II stage (7-10 months), according to the classification by Castro Leão (2009), which is an already consolidated stable developmental stage.
