# Supplementary material for: Prophylactic action of ayahuasca in a non-human primate model of depressive-like behavior
Source: Front Behav Neurosci. 2022 Nov 4;16:901425. doi: 10.3389/fnbeh.2022.901425 (PMC9672345; doi:10.3389/fnbeh.2022.901425)
Supplement: Supplementary file 2 [file Table_2.pdf]

Supplementary material

Table S2. Median and interquartile range ($M_d(Q_{25}, Q_{75})$) data at baseline and final week by groups.

		Familiar Control Group		Isolated Control Group		Ayahuasca Group	
		BP	FW	BP	FW	BP	FW
**Sucrose solution intake**	(s)	8.30 (0.29,60.5)	2.30 (0.0,52.0)	17.03 (1.5,47.0)	0.00 (0.0,0.5)	31.25 (10.0,55.1)	53.33 (41.0,94.7)
**Scent-mark**	(f)	1.00 (0.00,3.00)	1.00 (0.00,4.60)	2.33 (0.50,4.00)	4.00 (2.00,7.50)	1.00 (0.00,1.00)	1.00 (0.00,3.00)
**Autogrooming**	(s)	2.01 (0.00,29.75)	1,00 (0.00,19.80)	0,00 (0.00,4.25)	20.66 (0.00,110.71)	0,00 (0.00,4.37)	0.00 (0.00,0.25)
**Food Ingestion**	(s)	65,00 (25.96,102.60)	36.43 (7.59,69.90)	54.79 (17.50,82.00)	35.15 12.03,75.64)	189,00 (94.50,311.75)	58.50 (0.00,107.25)
**Locomotion**	(f)	24.00 (15.50,34.75)	23.00 (8.50,52.00)	29.00 (14.50,39.50)	30.00 (15.50,58.00)	18.00 (11.00,23.25)	35.00 (18.50,58.50)
**Scratching**	(s)	47.34 (23.91,67.53)	31.46 (16.58,57.00)	31.45 (24.63,46.38)	82.78 (52.62,118.26)	19.50 (5.00,42,75)	33.00 (5.00,69.37)
**Piloerection**	(f)	0,00 (0.00,0.00)	0.00 (0.00,0.00)	0,00 (0.00,0.00)	0.00 (0.00,0.00)	0,00 (0.00,0.00)	0.00 (0.00,0.00)
**Cortisol**	(ng)	2.97 (1.55,5.05)	4.24 (2.56,6.38)	2.10 (1.17,3.61)	1.69 (1.07,2.71)	0.23 (0.09,1.15)	0.64 (0.18,8.82)
**Age&**	(m)	7 (7,7)	-	7 (7,7)	-	8 (7.75,9)	-
**Weight#**	(g)	268 (226.75,274.5)	-	241.5 (237,257)	-	239 (236.5,247.5)	-

BP = baseline phase, FW = final week, s = seconds, f = frequency, m = months.

&: All animals were 7-9 months old, within the juvenile II stage (7-10 months), according to the classification by Castro Leão (2009).

#: No weight difference between groups was found (Kruskal-Wallis: H = 0.698, p = 0.706).
