# Supplementary material for: Prophylactic action of ayahuasca in a non-human primate model of depressive-like behavior
Source: Front Behav Neurosci. 2022 Nov 4;16:901425. doi: 10.3389/fnbeh.2022.901425 (PMC9672345; doi:10.3389/fnbeh.2022.901425)
Supplement: Supplementary file 3 [file Table_3.pdf]

Supplementary material

Table S3. Boruta's test for behaviors importance in group discrimination. I: mean variable importance; *: rejected variable for the comparison between groups.

**Parameters**	**(I)**
Scratching	3.075
Scent marking	-1.040*
Autogrooming	4.088
Food ingestion	2.913
Locomotion	0.754
Individual piloerection	-2.143*
Ingestion of the aqueous sucrose solution	11.231
