# Supplementary material for: Prophylactic action of ayahuasca in a non-human primate model of depressive-like behavior
Source: Front Behav Neurosci. 2022 Nov 4;16:901425. doi: 10.3389/fnbeh.2022.901425 (PMC9672345; doi:10.3389/fnbeh.2022.901425)
Supplement: Supplementary file 4 [file Table_4.pdf]

Supplementary material

Table S4. Median and quartiles ($M_d(Q25, Q75)$) for baseline behaviors considered important according to the Boruta algorithm, along with the respective significance test.

		Familiar Control Group	Isolated Control Group	Ayahuasca Group	H (p-value)	*Post-hoc* (p-value)
**Σ Sucrose solution intake**	(s)	58.1 (27.7,261)	129 (109,264)	284 (172,351)	2.78 (0.249)	-
**Σ Autogrooming**	(s)	61.5 (14.7,220)	14.3 (4.9,15)	14.6 (5.21,27.7)	2.73 (0.255)	-
**Σ Food Ingestion**	(s)	459 (400,609)	384 (254,407)	1480 (1088,1732)	11.3 (0.003)	AG > FG (0.007)<br>AG > IG (0.013)<br>FG = IG (0.927)
**Σ Scratching**	(s)	344 (267,419)	222 (214,233)	177 (115,265)	4.07 (0.131)	-

BP = baseline phase, FW = final week, s = seconds, f = frequency, m = months, Σ = sum of individual data, H = H-statistic (Kruskal-Wallis).
Despite the greater food intake in the ayahuasca group, it is not correlated with sucrose solution intake (Spearman: r = 0.66, p = 0.175).
All animals were 7-9 months old, within the juvenile II stage (7-10 months), according to the classification by Castro Leão (2009).
